# Ophthalmic Tethered Gold Yarnball‐Mediated Retained Drug Delivery for Eye Fundus Disease Treatment

**DOI:** 10.1002/smsc.202400095

**Published:** 2024-06-27

**Authors:** Shih‐Jie Chou, Yi‐Ping Yang, Min‐Ren Chiang, Chih‐Ying Chen, Henkie Isahwan Ahmad Mulyadi Lai, Yi‐Ying Lin, You‐Ren Wu, I‐Chieh Wang, Aliaksandr A. Yarmishyn, Guang‐Yuh Chiou, Tai‐Chi Lin, De‐Kuang Hwang, Shih‐Jen Chen, Yueh Chien, Shang‐Hsiu Hu, Shih‐Hwa Chiou

**Affiliations:** ^1^ Department of Medical Research Taipei Veterans General Hospital Taipei 112201 Taiwan; ^2^ Institute of Pharmacology College of Medicine National Yang Ming Chiao Tung University Taipei 112304 Taiwan; ^3^ Department of Biomedical Engineering and Environmental Sciences National Tsing Hua University Hsinchu 300044 Taiwan; ^4^ Department of Medical Sciences, Faculty of Health Sciences University College of MAIWP International Kuala Lumpur 68100 Malaysia; ^5^ Department of Biological Science and Technology National Yang Ming Chiao Tung University Hsinchu 300093 Taiwan; ^6^ Center for Intelligent Drug Systems and Smart Bio‐Devices National Yang Ming Chiao Tung University Hsinchu 300093 Taiwan; ^7^ Department of Ophthalmology Taipei Veterans General Hospital Taipei 112201 Taiwan; ^8^ School of Medicine National Yang Ming Chiao Tung University Taipei 112304 Taiwan; ^9^ Genomic Research Center Academia Sinica Taipei 115024 Taiwan

**Keywords:** age‐related macular degenerations, eye‐fundus diseases, gold nanoparticles, ocular drug deliveries, ophthalmic drug retentions

## Abstract

Eye fundus diseases, such as retinal degenerative diseases, which lead to blindness in ≈12% of individuals aged >65 years, cause permanent damage to retinal cells. The antioxidant quercetin (QC) is promising for the effective treatment of eye fundus diseases; however, its poor solubility and low retention rate often limit its clinical application. Herein, an in situ ophthalmic tethered gold yarnball (GY) that doubles as an ocular retention agent and QC reservoir to overcome low fundus drug retention is developed. After intravitreal injection, QC@GYs enhance retinal cell leakage and internal limiting membrane permeability, facilitating the partial penetration of QC@GYs into the intraretinal tissue. The combination of retina‐tethered QC@GY and first‐level sustained release reduces macular degeneration in vivo by effectively regulating oxidative stress. Furthermore, the sustained release of QC preserves the viability of retinal pigment epithelium cells, reduces apoptosis, and suppresses drusen formation. This preservation of retinal morphology and function maximizes the therapeutic impact while minimizing the need for frequent intraocular administration. Overall, the ophthalmic tethered GY platform is a versatile tool for retinal drug delivery for the treatment of eye fundus diseases.

## Graphical abstract

An in situ ophthalmic tethered gold yarnball (GY) that doubles as an ocular retention agent and quercetin (QC) reservoir to overcome low fundus drug retention is developed. After intravitreal injection, QC@GYs enhances retinal cell leakage and internal limiting membrane permeability, facilitating the partial penetration of QC@GYs into intraretinal tissue and sustained release of QC.

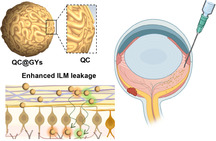

## 1 Introduction

Age‐related macular degeneration (AMD), which is primarily caused by retinal pigment epithelial (RPE) cell apoptosis in the macular area, is the most common cause of blindness in the elderly population.^[^ ^1^, ^2^ ^]^ Clinically, AMD is divided into wet AMD and dry AMD. Although wet (exudative) AMD is characterized by abnormal blood vessel formation, it can be treated with antivascular endothelial growth factor therapy.^[^ ^3^, ^4^ ^]^ However, dry AMD characterized by the accumulation of protein/lipid deposits (called drusen) under the retina has no effective treatment. In the late stage of dry AMD, geographic atrophy of the macular region in the advanced stages leads to severe central vision loss.^[^ ^5^, ^6^, ^7^, ^8^, ^9^, ^10^ ^]^ Furthermore, oxidative stress occurs due to an imbalance between the systemic manifestations of reactive oxygen species (ROS) and the ability of biological systems to detoxify or repair the damage caused.^[^ ^11^, ^12^, ^13^, ^14^ ^]^ The macular region of the retina having high metabolic activity is also a high source of oxidative stress.^[^ ^15^, ^16^ ^]^ Although intraocular injections facilitate the delivery of therapeutic drugs directly to the posterior segment of the eye using a hypodermic needle, the procedure carries risks of endophthalmitis, bulbar hemorrhage, and retinal detachment, especially with multiple injections.^[^ ^17^, ^18^, ^19^, ^20^, ^21^ ^]^

Quercetin (QC), a flavonoid that naturally occurs in numerous plant species, holds promise as a treatment for dry AMD because of its antioxidant and anti‐inflammatory properties.^[^ ^22^, ^23^, ^24^, ^25^ ^]^ However, the limited solubility of QC, along with its inadequate retention and targeting capabilities, significantly hinders its effectiveness in treating eye fundus diseases.^[^ ^26^, ^27^ ^]^ To address the ocular drug delivery, viscosity‐enhancing polymers and in situ gelling systems have been used to extend drug retention time in different eyedrop formulations and facilitate prolonged ophthalmic drug delivery.^[^ ^28^, ^29^, ^30^, ^31^ ^]^ However, these methods are primarily applicable to short retention and have limited enhancement in the intraocular penetration.^[^ ^32^ ^]^ Another less invasive method involving microneedles was originally developed for transdermal drug delivery and is now used to deliver drugs to the cornea and sclera. However, microneedling carries the risks of eye damage and potential infections. Recently, a polymeric nanomicelle system was developed for the delivery of macromolecular drugs for AMD treatment by extending their retention time in the eye. Although therapeutic efficacy has been demonstrated in mice with early choroidal neovascularization using high doses of antibodies, the mechanism of corneal penetration of this polymeric micelle system and its pharmacokinetics and dynamics in large eyes remain unclear.^[^ ^33^, ^34^ ^]^ Therefore, effective penetration and retention delivery of ophthalmic drugs to treat eye fundus diseases requires extensive exploration.^[^ ^35^ ^]^

Despite recent advances in ophthalmic drug delivery, achieving long‐term sustained release and low ophthalmic retention remains a significant obstacle in the treatment of eye fundus diseases, such as AMD.^[^ ^36^, ^37^, ^38^, ^39^ ^]^ Difficulties arise from complex barriers in the eye, undesired release, and insufficient drug exposure of target tissues.^[^ ^18^, ^40^, ^41^, ^42^ ^]^ To modulate tissue microenvironments, researchers have documented the effects of nanoparticle (NP) densities and sizes on NP‐induced endothelial leakiness (nanoEL), which can disrupt and release cell biointeractions.^[^ ^43^, ^44^, ^45^, ^46^ ^]^ Among these particles, gold NPs (AuNPs) possessing high biocompatibility have been observed in all layers of the retinal structure in vivo, indicating the penetration of the blood–retinal barrier.^[^ ^47^, ^48^, ^49^ ^]^ Furthermore, AuNPs functionalized with hyaluronic acid have demonstrated the capability to cross anatomical barriers and access retinal and choroidal regions. Recent studies have also suggested that high‐density gold or inorganic NPs in tissues can disrupt cell–cell interactions, potentially facilitating drug delivery.^[^ ^40^, ^44^, ^45^, ^50^, ^51^ ^]^ Furthermore, the small size and unique features of textured porous gold nanoparticle (GNP) enable efficient drug loading and delivery to target sites.^[^ ^14^, ^52^, ^53^ ^]^

**Figure 1:**
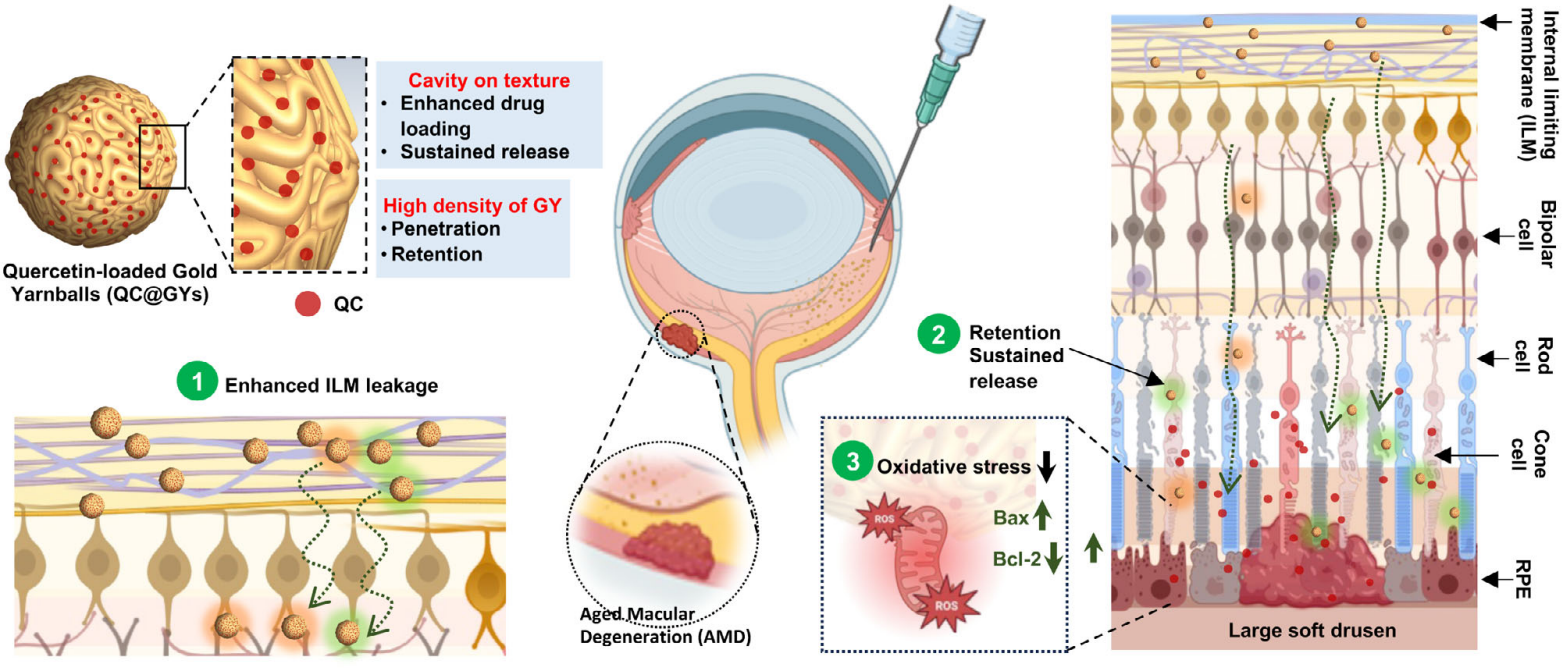
Schematic illustration of QC@GY for efficient delivery of poorly soluble drugs to treat fundus diseases. The sustained release of QC preserves RPE cell viability, reduces apoptosis, and suppresses drusen formation.

In the present study, QC‐loaded gold yarnball (QC@GY), that combines the features of enhanced ocular retention and sustained drug release, was developed (**Figure** **1**). Using a seed growth method, GY was found to possess a distinctive cavity texture that enables efficient loading of QC and facilitates sustained release. Following the intravitreal injection of QC@GYs, the presence of GYs enhanced the permeability of the internal limiting membrane (ILM), facilitating the partial penetration of QC@GYs into the deep intraretinal tissue. Ophthalmic site‐tethered GYs combined with a first‐order sustained release demonstrated promising efficacy in mitigating oxidative stress‐induced AMD by modulating oxidative stress. Moreover, the sustained release of QC preserved the viability of RPE cells, reduced apoptosis, and suppressed drusen formation. This preservation of retinal morphology and function maximizes the therapeutic effect while minimizing the need for frequent intravitreal administration. This ophthalmic tethered GY provides a multifunctional nanotechnology for sustaining ophthalmic drug delivery in the treatment of eye fundus diseases.

## 2 Results

### 2.1 Preparation and Physicochemical Characterization of QC‐Conjugated GY (QC@GY)

**Figure 2:**
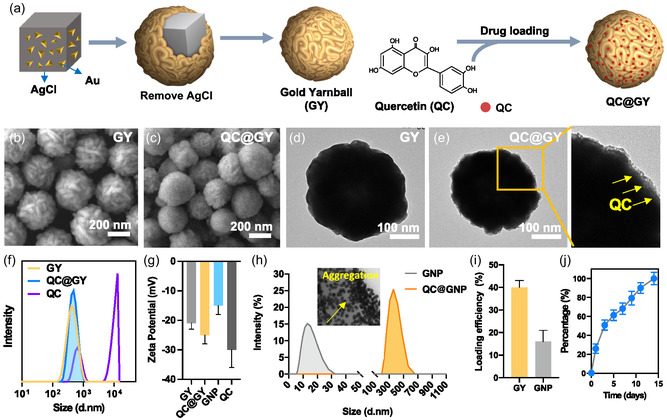
Preparation and physicochemical characterization of QC‐conjugated GY (QC@GY) NPs. a) Schematic illustrating of synthesis the QC@GYs. b,c) SEM images of GYs (left panel) and QC@GYs (right panel). d,e) TEM images of GYs, QC, and QC@GYs. Right panel: Magnified image with yellow arrows showing bound QC. f) Size distribution of GY, QC, and QC@GY. Unloaded QC showing aggregation in water solution. g) Measurement of zeta potentials of GYs and GNPs, their QC‐loaded forms (QC@GY and QC@GNP) and free QC. h) Size distribution of GNP and QC@GNP. A scheme illustrating GY's better performance than GNP for drug release and delivery. i) Loading efficiency of QC to GYs as compared to GNPs. j) The release pattern of QC from QC@GYs. Numerical data from (f) to (j) are mean values with SD, *n* = 3.

GYs were synthesized using the seed‐growth method following established procedures reported in previous studies (**Figure** **2**a).^[^ ^54^ ^]^ Gold (Au) ligaments were meticulously grown on the surface of cubic silver chloride (AgCl) and employed as sacrificial templates. The formation of cubic AgCl was facilitated using polyvinylpyrrolidone (PVP) as a capping agent, which was achieved by exchanging silver nitrate ions with ethylene glycol. Subsequently, gold seeds are deposited within the defects of the AgCl cube, resulting in a low coordination number and high affinity. During the synthesis process, a covalent bond was established between the surfaces of the GNPs in AgCl and the GNP. Hydroquinone reduction contributed to the rapid formation of GYs with high densities of gold ligaments within a few minutes. The reduction in hydroquinone concentration would also influence the sizes and morphologies of the particles (Figure S1, Supporting Information). Ammonia solution was then introduced to dissolve the AgCl cubes. A distinct brownish color in the solutions after Ag/AgCl loading indicated the generation of surface plasma by GY, whereas a pale yellow color emerged upon GY integration. Concurrent with GY synthesis, drug coating of the NPs was observed. The GNP‐QC complexes (QC@GY) were formulated by adding varying amounts of QC (1 mg mL^−1^ in DMSO) to a mixture of chloroauric acid and trisodium citrate to achieve the desired drug concentration during boiling.

Scanning electron microscopy (SEM) images of the GYs showed a spherical NP morphology with an average diameter of 300 nm (Figure 2b). The unique ligament surface of GYs, characterized by a thickness and length of ≈17 ± 3 and 50 ± 18 nm, respectively, was successfully prepared. In contrast, AuNPs are notably smaller, with diameters of <10 nm. Loading QC resulted in a smoother NP surface, which was attributed to the QC filling the pores between the gold ligaments (Figure 2c). Transmission electron microscopy (TEM) confirmed a uniform layer of loaded QC on the surface of QC@GYs (Figure 2d,e). Remarkably, despite loading the highly hydrophobic QC drug, there was no apparent particle aggregation, as evidenced by dynamic light scattering analysis (Figure 2f). Zeta potential measurements indicated a decrease in the negative charge on the surface of free GYs from −20 to −23 mV after QC loading (Figure 2g).

To compare the loading of poorly soluble drugs between the 5 nm AuNPs and GY, an equivalent drug capacity was loaded onto the GNP. However, Figure 2h shows significant aggregation upon using 5 nm GNP. This observation suggests that the GY cavity is crucial for accommodating large amounts of poorly soluble drugs. These favorable drug‐carrying results may stem from hydrophobic interactions between the drug molecules and the metal surface of GY. Moreover, GY has an increased surface area, providing numerous sites for drug molecules to adhere to, thereby enhancing particle loading capacity. Additionally, GY facilitates the dissolution of poorly soluble drug molecules by creating a conducive environment for interactions with solvents or biological fluids. Consequently, this feature can improve drug release kinetics and bioavailability.

Quantification of the QC loading efficiency revealed ≈40% for GYs, twice as high as that for GNPs, aligning with the unique ligamentous porous structure of the former and significantly increasing the binding surface (Figure 2i). Furthermore, the loaded QC exhibited sustained‐release properties from the GYs for at least 15 d (Figure 2j). These results confirm the capability of GYs to stably load drugs, demonstrating their potential as effective drug delivery systems. The GY cavities provide a network of interconnected voids or channels within the particle structure. These pores serve as reservoirs for loaded drug molecules, allowing for controlled release over time. Drug molecules loaded within the pores undergo diffusion‐controlled release. As the surrounding medium penetrates the pores, it comes in contact with the drug molecules, allowing them to gradually diffuse.

### 2.2 Internalization of QC@GYs Mediates Protective Antioxidant Effect on Human RPE Cells

**Figure 3:**
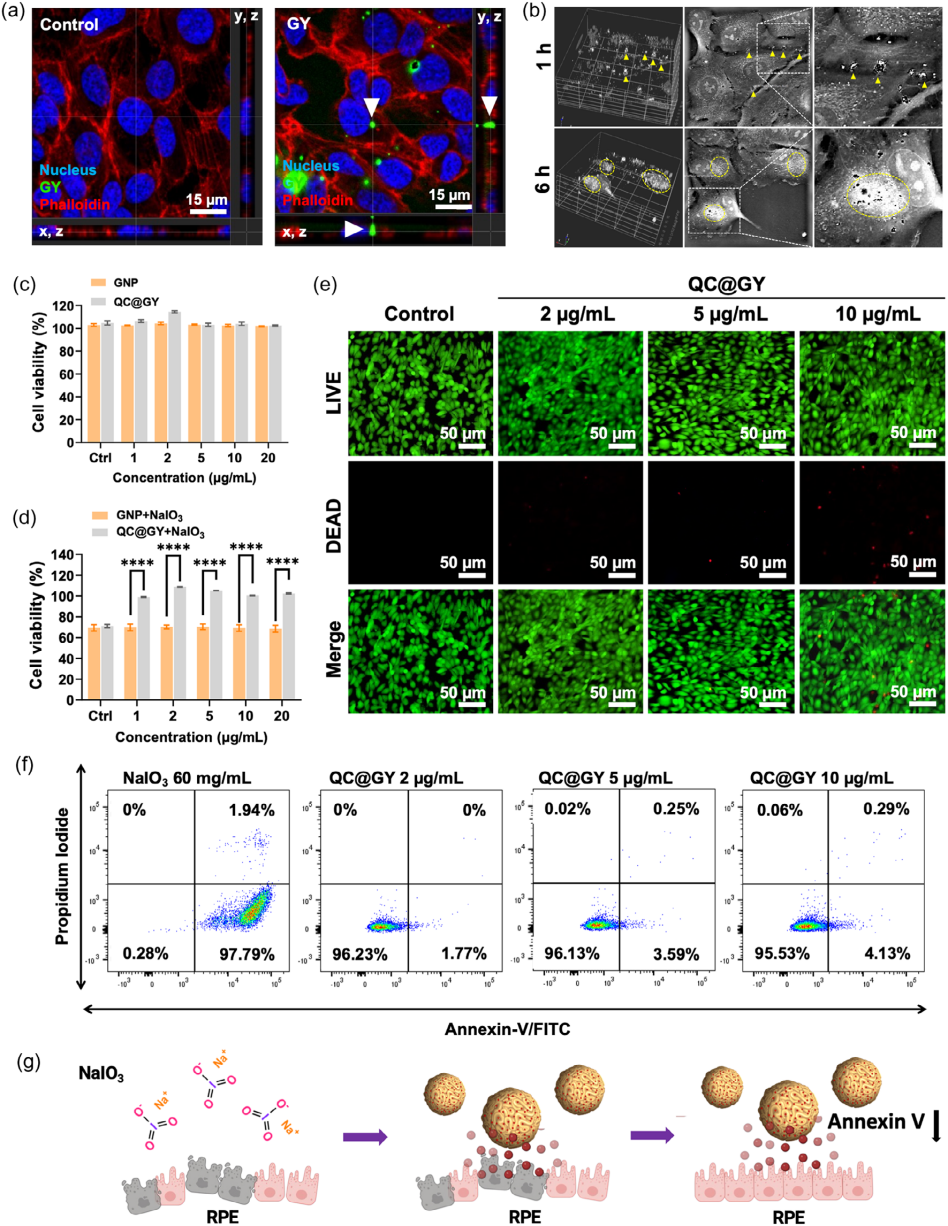
Internalization of QC@GYs mediates protective antioxidant effect on human RPE cells. a) Confocal images demonstrate the effective permeation and disruption of cellular TJs by GY. The arrowhead indicates the intracellular location of GY. The image axes are labeled (*x*,*y*, *y*,*z*, and *x*,*z*). b) Holotomography images of ARPE‐19 cells after incubation with QC@GY. Yellow arrowheads indicate QC@GY, yellow dashed circles indicate localization clusters of QC@GY. c) Alamar blue viability assay showing the effect of the indicated concentrations of GY and GC@GY NPs on the viability of ARPE‐19 cells. Mean relative values are shown with ±SD, *n* = 3. d) Cell viability for NaIO_3_‐treated ARPE‐19 cells in the absence or presence of GY or QC@GY GY by the Alamar blue assay. The data were expressed as mean ± SD, *n* = 3; ****p < 0.0005. e) LIVE/DEAD assay fluorescent images of ARPE‐19 cells treated with the indicated concentrations of QC@GY NPs. Green fluorescence indicates live cells, red fluorescence indicates dead cells. f) Flow cytometry analysis of annexin V/FITC‐stained ARPE‐19 cells subjected to the treatment with NaIO_3_ and the indicated concentrations of QC@GY. g) A scheme illustrating the protective effect of QC@GY against NaIO_3_‐injured RPE cells.

Internalization of GY NPs is critical for promoting protective antioxidant effects in human RPE cells. To assess internalization, GYs were incubated with ARPE‐19 cells. The confocal laser scanning microscopy (CLSM) image in **Figure** **3**a shows strong internalization of GY. Internalization of NPs can be attributed to passive cellular uptake mechanisms, including diffusion, endocytosis, and phagocytosis. After entering the cells, these NPs tended to accumulate near the perinuclear region, possibly because of the abundance of cellular structures. These organelles play a key role in intracellular trafficking and processing. Instant holographic microscopy was used to evaluate the 3D morphology and refractive index, as shown in Figure 3b. The results showed intercellular leakage after 1 and 6 h of co‐culture. This observation is reminiscent of NP‐induced endothelial leakage (nanoEL), in which exposure to specific NPs results in increased cell permeability. This increased permeability causes fluids and molecules to leak from the bloodstream into surrounding tissues.^[^ ^43^, ^44^, ^45^, ^46^ ^]^ These effects were further confirmed using a Transwell assay in Figure S2, Supporting Information. Following a 24 h culture of compact cells in the upper layer with GYs, the presence of particles was observed in the bottom cells, signifying an enhancement in cell sheet permeability.

To examine whether QC@GY were cytotoxic, ARPE‐19 cells were treated with various concentrations of QC@GY for 24 h and an AlamarBlue assay was used to assess ARPE‐19 cell viability. The viability of ARPE‐19 cells was not affected following the treatment even with QC@GY concentration as high as 20 μg mL^−1^ (Figure 3c). Next, an in vitro cell model was established, and oxidative stress conditions were reproduced by treatment with NaIO_3_, a potent inducer of oxidative stress that can selectively damage RPE cells, as previously described.^[^ ^55^ ^]^ Oxidative stress‐induced RPE cell death is a key factor in dry AMD progression. Evaluation of antioxidant effects of QC@GY in NaIO_3_‐treated ARPE‐19 cells was done. The cells were treated with 20 mM m NaIO_3_ at various concentrations of GY or QCs@GY for 24 h, and an AlamarBlue cell viability assay was performed. Compared with GY, QC@GY significantly improved the viability of NaIO_3_‐treated ARPE‐19 cells. Furthermore, an increase in QC@GY concentration did not result in an improvement in cell viability in the presence of NaIO_3_, indicating that a concentration of 1 μg mL^−1^ is sufficient to prevent the effects of NaIO_3_‐induced oxidative stress (Figure 3d,e).

To determine whether QC@GY treatment exerted an antiapoptotic effect under oxidative stress, ARPE‐19 cells were stained with Annexin V‐FITC/PI after cotreatment with 20 mM NaIO_3_ and different concentrations of QC@GY for 24 h. As shown by flow cytometry, treatment with NaIO_3_ alone drastically increased the percentage of Annexin V‐stained cells (97.79%; *p* < 0.05). However, cotreatment with QC‐GY NPs markedly reduced the proportion of annexin V‐stained cells, indicating suppression of apoptosis by QC‐GY NPs (Figure 3f,g). In summary, we demonstrated that QC@GY generally has no cytotoxicity and can be internalized to alleviate oxidative stress‐induced insults in RPE cells.

### 2.3 QC@GYs Prevent AMD‐Related Histopathological Changes in the Mouse Retinas Treated with NaIO_3_

**Figure 4:**
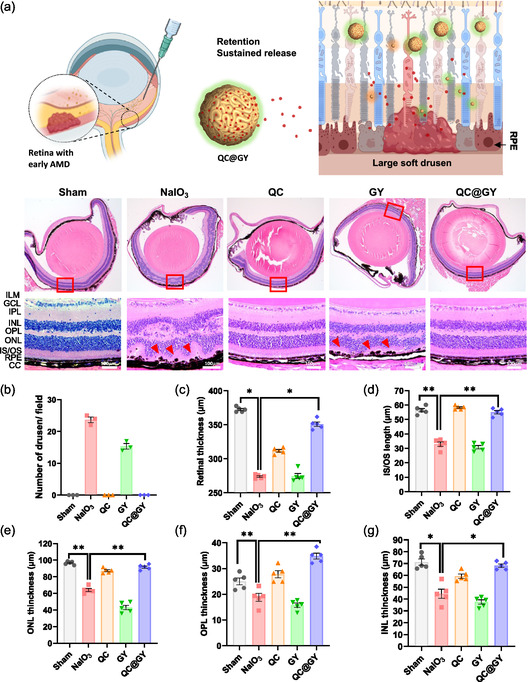
The protective effect of QC@GYs on the mouse retina structure and function exposed to NaIO_3_‐induced oxidative stress. a) Representative images of H&E staining of the mouse eyes cross sections treated as indicated. Top panel: whole eye cross sections. Bottom panel: magnified retinal and choroidal regions, ILM, retinal GCL, IPL, INL, OPL, ONL, IS/OS layer of photoreceptors, RPE layer, BM, and CC layer, shown within red rectangles in the top panel. Red arrows indicate drusen deposits. b–g) Quantification of the drusen numbers and the thickness of indicated retinal layers: number of drusen deposits (b), retinal thickness (from the ILM to CC); (c), photoreceptor IS/OS length (d), ONL thickness (e), OPL thickness (f), and INL thickness (g). Mean values with SD error bars are shown, *n* = 3, *p < 0.05, **p < 0.01.

NaIO_3_ administration has been widely used to model dry AMD in vivo because it induces reproducible and selective retinal degeneration.^[^ ^56^, ^57^, ^58^ ^]^ To evaluate the in vivo effects of QC@GYs, we used a mouse model of AMD, as described in our recent study.^[^ ^59^ ^]^ QC@GY (100 mg kg^−1^) was intravitreally injected into C57BL/6 J mice, followed by intraperitoneal injection of 30 mg kg^−1^ NaIO_3_ the following day to induce intraocular oxidative stress (**Figure** **4**a). Seven days after induction and treatment, the mice were euthanized and their eyes were subjected to histological examination using hematoxylin and eosin (H&E) staining. H&E staining showed that NaIO_3_‐induced retinal injury was manifested by significant distortion of all retinal and choroidal structures, including the ILM, retinal ganglion cell layer (GCL), inner plexiform layer (IPL), inner nuclear layer (INL), outer plexiform layer (OPL), outer nuclear layer (ONL), inner segment/outer segment (IS/OS) layer of photoreceptors, RPE layer, Brush's membrane (BM), and choriocapillaris (CC) layer. (Figure 4a). Through this measurement, we were able to evaluate the effect of NaIO_3_‐induced insults on the thickness of all retinal and CC layers that were affected by NaIO_3_ in previous studies.^[^ ^60^, ^61^ ^]^ In addition, several bulging formations resembling drusen deposits, a typical feature of dry AMD, emerged from the RPE layer. QC alone only slightly decreased drusen deposition. Similar to the intact retina of the sham control mice, pretreatment with QC@GYs preserved the retinal structure despite NaIO_3_ treatment. This treatment completely prevented the formation of the drusen deposits (Figure 4b). As quantified using ImageJ software, the average thickness of the retina at the posterior part of the eyeball (Figure 4c), IS/OS length (Figure 4d), ONL thickness (Figure 4e), OPL thickness (Figure 4f), and INL thickness (Figure 4g) were significantly reduced by NaIO_3_ treatment. QC alone had a moderate effect on the improvement of these parameters. Administration of QC@GYs significantly preserved these parameters, resembling those of the sham control mice. In summary, QC@GY administration effectively prevented the typical pathological features observed in the NaIO_3_‐injured mouse model of dry‐type AMD in vivo.

### 2.4 QC@GYs’ Decreasing Apoptosis and Inflammatory Response in the Retina Exposed to Oxidative Stress

To assess the retention and penetration dynamics of GYs, the particles were administered via intravitreal injection to the mice. After 24 h, the eyes were harvested, and the cell nuclei along with RGCs were stained (Figure S3, Supporting Information). Using depth profiling scanning with CLSM, minimal particle presence was noted in the superficial layer of the retina. However, distinct particle signals were evident at depths of 5 and 15 mm. These findings underscore the successful penetration and sustained retention of particles within the ocular tissues.

**Figure 5:**
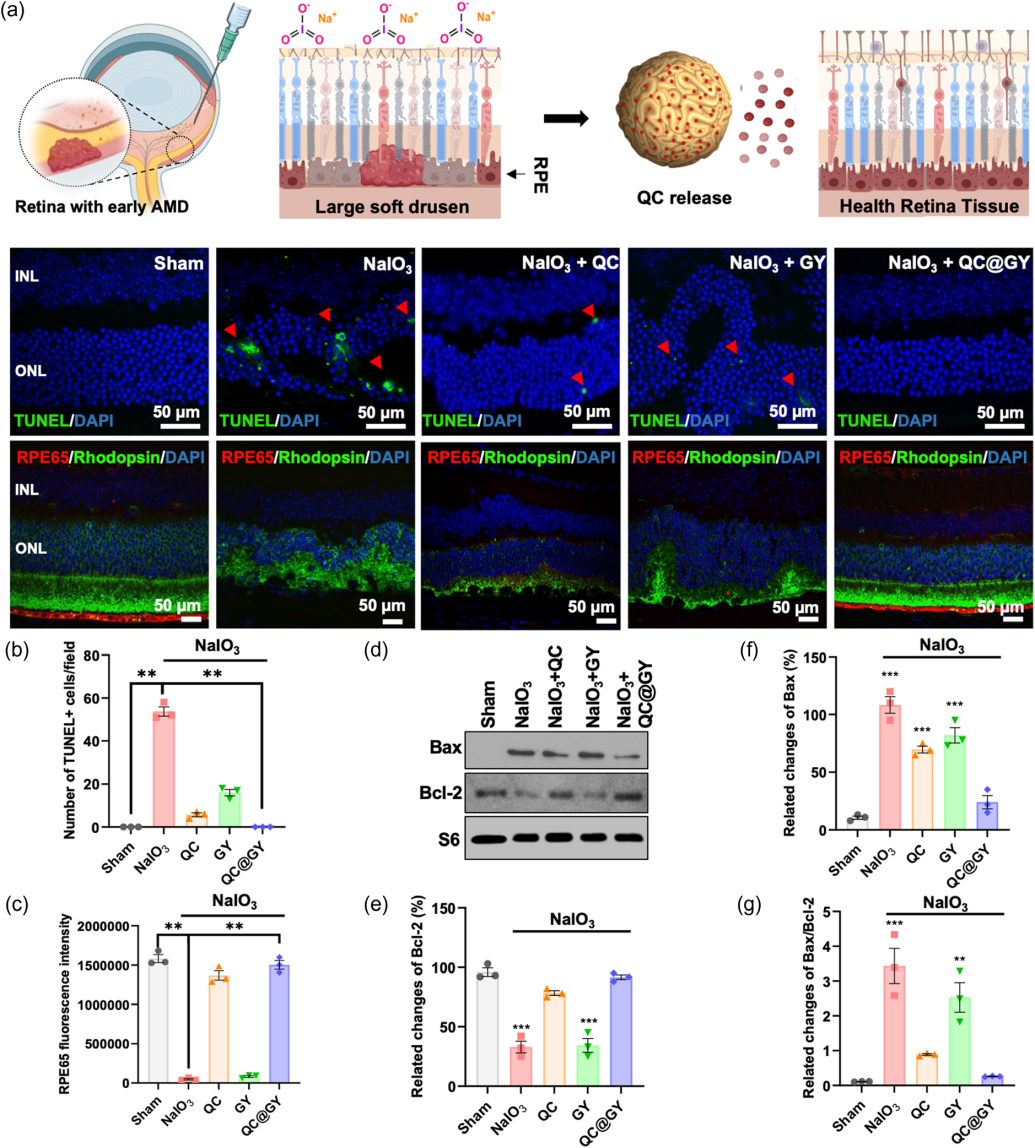
QC@GY decreases apoptosis in the retina exposed to oxidative stress. a) Representative fluorescent images of TUNEL staining (top panel) and immunostaining of RPE65 (RPE marker) and rhodopsin (photoreceptor marker) of the mouse retinal cross sections treated as indicated. Red arrowheads indicate TUNEL‐positive cells. Nuclei stained with DAPI. INL, inner nuclear layer and ONL, outer nuclear layer. b) Quantification of the number of TUNEL‐positive cells in panel A. c) Quantification of RPE65 fluorescence intensity in immunostaining images. Quantitative data in (b and c) were presented as means ± SD, *n* = 3, **p < 0.01. d) The representative Western blot images show the apoptotic protein levels in mouse retinas treated as indicated. S6 protein loading control. Western blot densitometry quantification of the expression of e) Bax and f) Bcl2. g) The Bax/Bcl2 ratio was used as the index for the susceptibility of the cells to apoptosis. Quantitative data in (e–g) were expressed as mean ± SD, *n* = 3, **p < 0.01 and ***p < 0.005.

Apoptosis is the key mechanism underlying retinal degeneration in NaIO_3_‐injured mouse dry‐type AMD model. Therefore, we conducted a dUTP nick end labeling (TUNEL) assay to evaluate whether QC@GY decreased NaIO_3_‐induced apoptosis in dry type AMD in vivo model. Treatment with NaIO_3_ markedly increased the number of TUNEL‐positive apoptotic cells and caused considerable deformation of the retinal layers by decreasing the number of retinal cells compared to the sham control (**Figure** **5**a,b). QC treatment alone slightly reduced the number of TUNEL‐positive apoptotic cells. Concomitant treatment with QC‐GY prevented apoptosis and retinal deformation Furthermore, we aimed to identify the retinal cell type that was primarily affected by the oxidative damage induced by NaIO_3_. Immunostaining of RPE cells and photoreceptors with RPE65 antibodies and rhodopsin, respectively, revealed complete degradation of the RPE layer and considerable deformation of the photoreceptor layer in NaIO_3_‐treated mice (Figure 5c). Upon NaIO_3_ treatment, the administration of GY alone slightly decreased the number of TUNEL‐positive cells, whereas the administration of unloaded QC showed a moderate suppression of TUNEL‐positive apoptotic cells. Importantly, the NaIO_3_‐injured mice treated with QC@GYs displayed normal RPE and retinal morphology, comparable to that of the sham control group.

To verify the mechanism of the QC@GY‐mediated alleviation of retinal apoptosis in NaIO_3_‐treated mice, we evaluated the protein expression levels of key regulators of apoptosis using Western blotting (Figure 5d). NaIO_3_ treatment upregulated the proapoptotic factor Bax, whereas concomitant treatment with QC@GYs efficiently prevented Bax upregulation. Furthermore, our data showed that the level of the antiapoptotic protein Bcl‐2 was not affected by either NaIO_3_ treatment or concomitant treatment with QC@GY. The ratio of Bax to Bcl2 was used to determine susceptibility to cellular apoptosis.^[^ ^60^ ^]^

**Figure 6:**
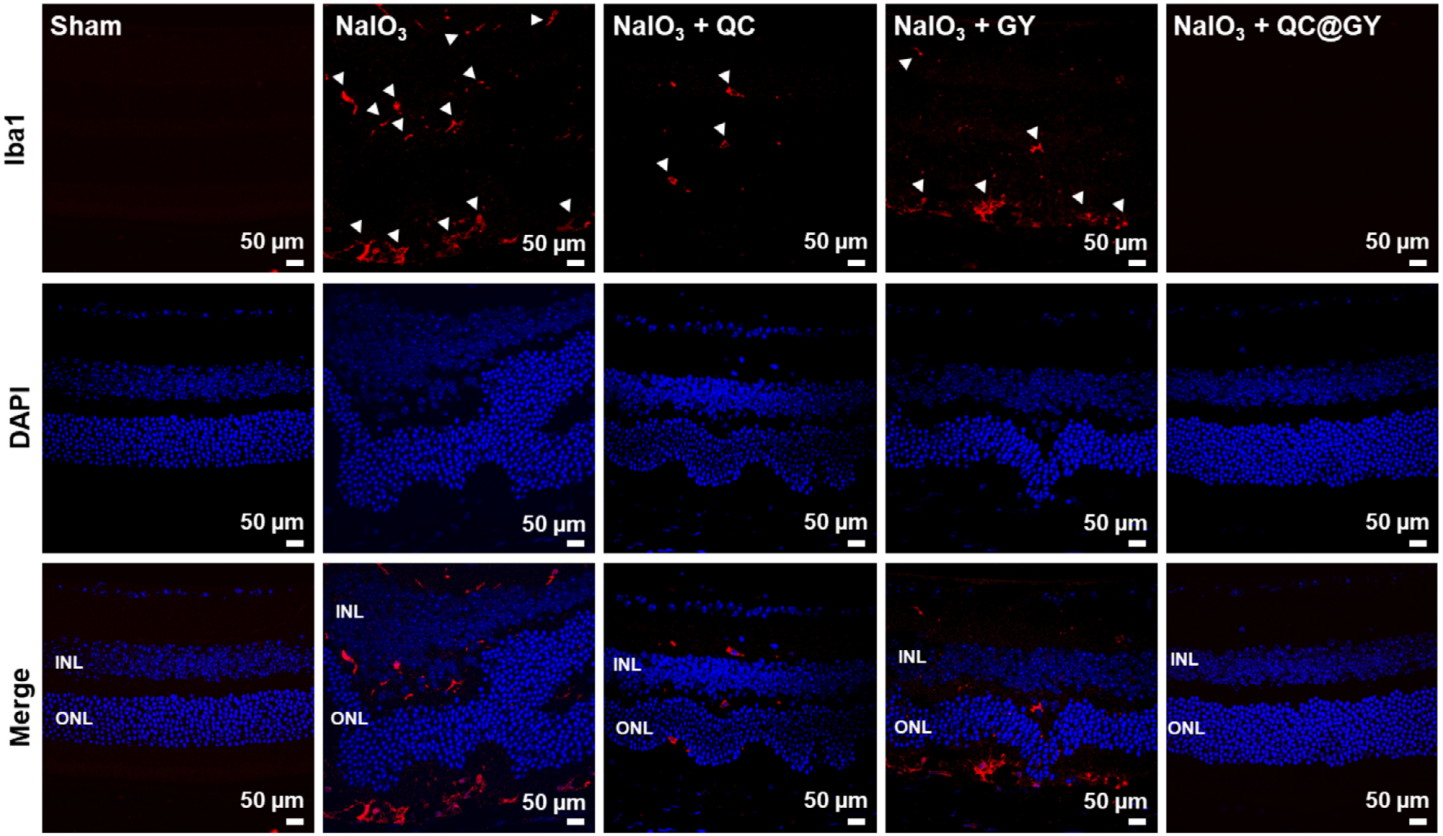
Immunostaining of Iba1 microglial marker in the mouse retinal cross sections treated as indicated. White arrowheads indicate Iba1 signal.

Upon challenge with NaIO_3_, the Bax/Bcl‐2 ratio significantly increased. However, the Bax/Bcl‐2 ratio was significantly suppressed by the addition of free, unloaded QC, or QC@GY (lower right panel in Figure 5e). The pathophysiological features of dry AMD are closely associated with microglia‐mediated neuro‐inflammation.^[^ ^62^ ^]^ Therefore, we immunostained for ionized calcium‐binding adapter molecule 1 (IBA1), a marker of microglial cells. In NaIO_3_‐treated retinas, IBA1‐positive cells infiltrated different retinal layers, including the INL and ONL (**Figure** **6**). QC alone mildly reduced IBA1‐postive cell infiltration. In contrast, treatment with QC@GYs prevented the infiltration of IBA1‐positive microglia, which also correlated with the normal morphology of the retina (Figure 6). These findings suggest that QC@GYs can effectively prevent oxidative stress‐induced insults to the retinal structure by preventing the apoptosis of RPE cells and photoreceptors and alleviating neuroinflammatory responses.

### 2.5 Conjugation with GY Increases the Retention Time of QC in the Retina

**Figure 7:**
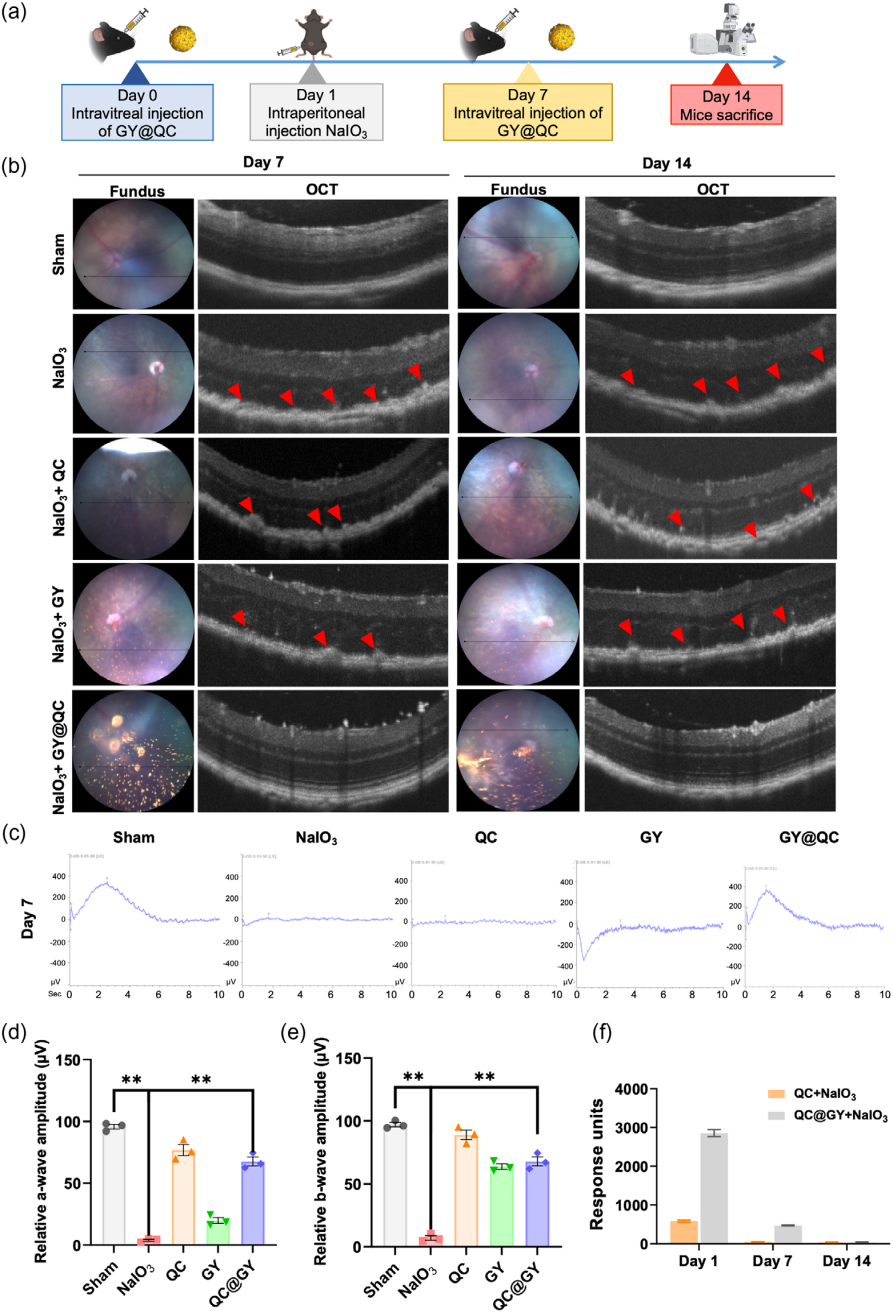
Conjugation with GYs enhances the retention time of QC in the retina, thereby improving therapeutic efficacy. a) Schematic diagram illustrating the experimental design to the study the sustain release of QC by QC@GY and its effect. b) Left panel: fundus images showing QC@GY internalized by the retina. Right panel: OCT images of the retinas exposed to the indicated treatments. Red arrowheads indicate drusen deposits. c–e) ERG measurements of eye electrophysiology revealed improved vision recovery following treatment with QC@GYs. The data were represented as mean ± SD, *n* = 3; **p < 0.01. f) LC‐MS measurement of QC levels in the NaIO3‐injured retinas treated with QC or QC@GYs.

Given that QC exhibited sustained release after conjugation with GYs in vitro, we evaluated the retention time of QC in the mouse eyes after GC@GY administration. QC@GYs and QC (100 mg kg^−1^) were intravitreally injected into the mice on day 0, followed by an intraperitoneal injection of 30 mg kg^−1^ NaIO_3_ the following day (**Figure** **7**a). After administration at onset, the injection of either QC or QC@GYs was repeated on day 7. The effects of QC and QC@GYs on NaIO_3_‐injured eyes were monitored using eye fundus photography and optical coherence tomography (OCT) in parallel, and the electrophysiological functions of the retinas were assessed using electroretinography (ERG). The vitreous humor samples were simultaneously collected on days 1, 7 and 14 and subjected to liquid chromatography‐mass spectrometry (LC‐MS) analysis to measure the released QC. Furthermore, a pharmacokinetic study using QC loaded with Cy5.5 was performed to evaluate the release of QC in vivo (Figure S4, Supporting Information). The in vivo imaging system (IVIS) results showed that the fluorescence intensity could be maintained after the first day of injection, which was consistent with previous data, indicating that QC could sustain drug release for at least 7 d. As observed by eye fundus photography, the QC@GY particles remained visible by day 14 after intravitreal injection. As evaluated by OCT, NaIO_3_ treatment resulted in considerable damage to the retina, with accumulation of drusen deposits on postinjection days 7 and 14 (Figure 7b). Concomitant administration of unloaded QC prevented retinal damage and decreased drusen formation on day 7; however, this effect was not observed by day 14. In contrast, the concomitant administration of QC@GYs resulted in the preservation of normal retinal structure without the formation of drusen by days 7 and 14 (Figure 7b). Additionally, we examined the effects of QC@GYs and QC on retinal electrophysiological function in NaIO_3_‐injured mice using ERG. Compared with the sham control mice, the amplitudes of both the a‐wave (Figure 7c,d) and b‐wave (Figure 7c,e) were significantly decreased in the NaIO_3_‐treated group. GY alone slightly increased the amplitudes of both waves, whereas QC and QC@GY both led to a robust increase in the amplitudes of the a‐ and b‐waves (Figure 7c,d). Compared with QC alone, the long‐acting efficacy of QC@GYs could be attributed to the conjugation of QC to GYs and the resultant sustained release. To verify this speculation, we measured and compared the amount of QC in vitreous humor samples with the indicated treatments on different postinjection days. On postinjection day 1, QC levels in the vitreous humor samples of QC@GY‐treated retinas were 6‐fold higher than those in the QC‐treated retinas (Figure 7f). On postinjection day 7, QC levels dropped to baseline in QC‐injected retinas, whereas they remained high in QC@GY‐injected retinas. Vitreous QC in QC‐GY‐injected retinas gradually declined to undetectable levels by day 14 (Figure 7f). Overall, compared with unloaded QC, the administration of QC@GY resulted in a longer retention time in the vitreous humor due to sustained QC release from the QC@GY complex and QC‐mediated beneficial efficacy against NaIO_3_‐mediated insult.

### 2.6 Long‐Term Follow‐Up Studies of QC@GYs Show No Systemic Adverse Effects In Vivo

**Figure 8:**
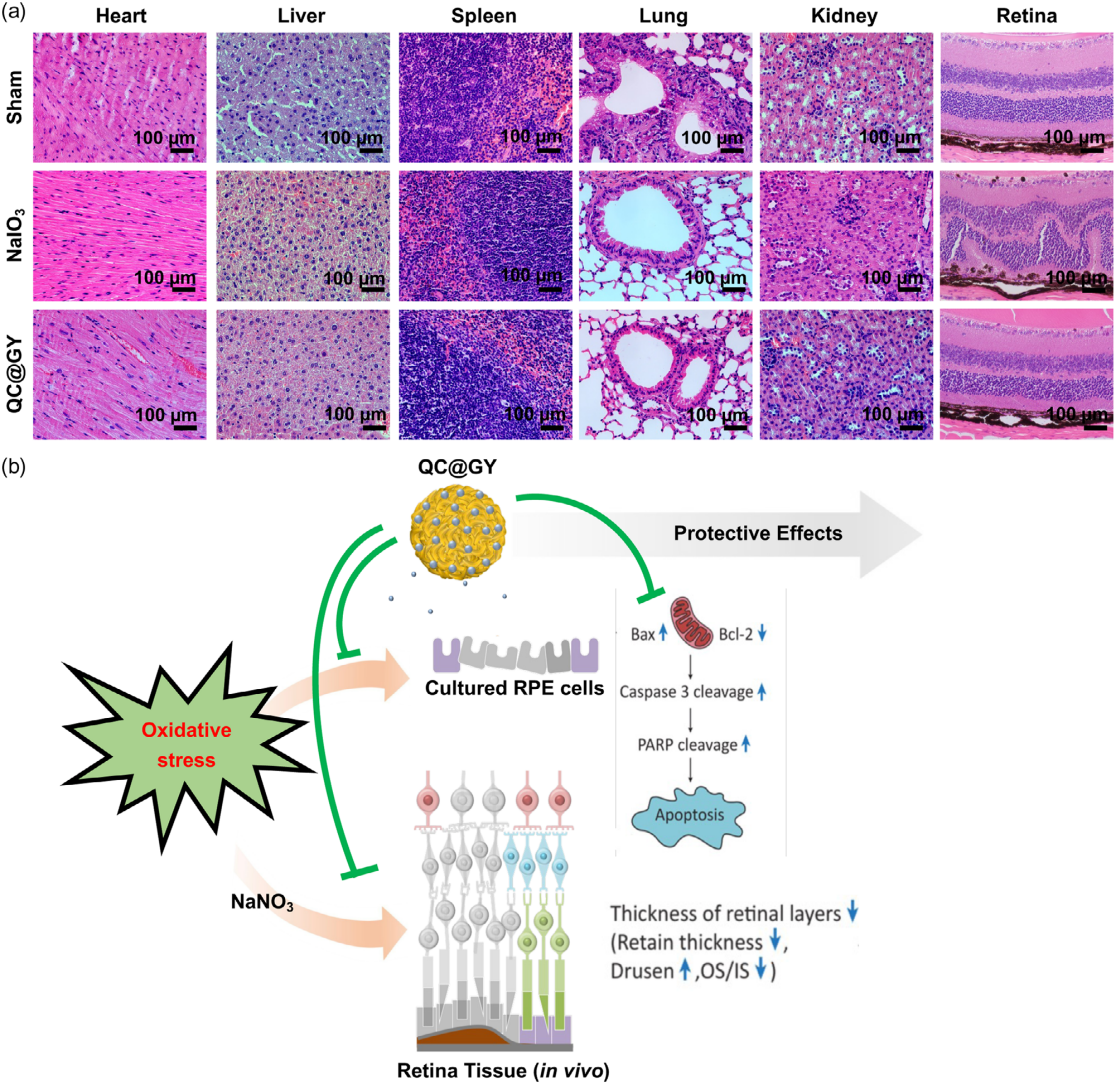
A representative scheme showing the protective effects of QC@GY against oxidative stress‐induced insult in vitro and in vivo. a) Representative H&E‐stained images of major organs; heart, liver, spleen, kidney, and retina from mice in the control, SI, and QC@GY group 7 days after treatments. Pathological damages were not observed in liver, spleen, kidney, heart, and lung. b) The scheme of QC@GY‐mediated protective effects against oxidative stress‐induced apoptosis and retinal damage in vitro and in vivo.

Toxicity studies that evaluate the in vivo biosafety of new drugs or molecules are crucial for conducting further biological studies. Therefore, we assessed the systemic toxicity of QC@GYs on postinjection day 14 in NaIO_3_‐treated mice (**Figure** **8**a). Compared to the Sham group, NaIO_3_ administration specifically led to damage to the retinal architecture and RPE layer without affecting other major organs (Figure S5, Supporting Information). Overall, QC‐GY ameliorated NaIO_3_‐induced retinal damage without unfavorable adverse effects. The administration of QC@GY did not cause any behavioral alterations in the mice, such as aggressiveness, tremors, respiratory distress, sleep, or coma. Pathological examination of major organs (heart, liver, spleen, lungs, and kidneys) revealed no degenerative, inflammatory, vascular, necrotic, or apoptotic lesions. Collectively, these data demonstrate that the long‐term use of QC@GYs carries no systemic toxicity (Figure 8b).

To confirm the biocompatibility of QC@GYs in the ocular tissue, we intravitreally injected QC@GYs into the eyes of mice. After 1 and 7 d, eye tissues were harvested and evaluated by H&E staining and terminal deoxynucleotidyl transferase TUNEL assays (Figure S5, Supporting Information). H&E staining revealed that the retinal structure remained intact and undamaged. In addition, TUNEL assay showed no apoptosis after treatment of QC@GYs, proving the safety of QC@GYs. The results showed biocompatibility data for eye tissue.

## 3 Discussion

Oxidative stress has been widely recognized as a major factor in AMD pathogenesis, and oxidative stress‐induced RPE cell death can lead to other types of retinal damage, including photoreceptor degeneration and thinning of multiple retinal layers. Owing to its excellent antioxidant potential, QC has shown multiple benefits in the treatment of dry AMD.^[^ ^63^, ^64^, ^65^, ^66^, ^67^ ^]^ However, the in vivo instability of QC and limited drug retention due to poor water solubility have largely challenged its clinical translation.^[^ ^68^ ^]^ Nanotechnology is one of the directions for developing drug delivery systems that meet clinical needs.^[^ ^69^ ^]^ This study provides compelling evidence that innovative QC nanocarrier formulations encapsulated within GY can effectively address the challenges stemming from the low aqueous solubility of this therapeutic agent. Moreover, QC@GY demonstrated prolonged drug release along with notable antiapoptotic and anti‐inflammatory effects in both in vitro and in vivo AMD models.

Although GY and GNP had similar physical properties, we observed that the QC loading capacity of GY was significantly higher than that of GNP. In fact, the “yarnball”‐like morphology of GY provides a higher surface area for drug loading.^[^ ^70^, ^71^ ^]^ GNPs have been shown to exert antioxidant activity both in vitro and in vivo.^[^ ^72^, ^73^ ^]^ The additive capping of GNPs with QC has also been shown to exhibit^[^ ^74^ ^]^ a higher antioxidant potential than free QC.^[^ ^75^, ^76^ ^]^ With a superior loading capacity of QC into GYs compared to GNPs, our findings demonstrate that QC@GYs effectively mitigated the NaIO_3_‐induced damage in RPE cells, both in vitro and in vivo. The protective action of QC@GYs was attributed to the antioxidative, antiapoptotic, and anti‐inflammatory properties of QC upon its release from GYs.

Efficient delivery requires sustained release of certain drugs at a programmed rate for a prolonged period.^[^ ^77^ ^]^ Manipulation of various biocompatible nanomaterials facilitates the controlled and extended release of drugs. QC is well known for its ability to scavenge free radicals and act as a chelating agent by inactivating iron ions that generate ROS.^[^ ^72^, ^75^ ^]^ In the present study, we constructed a GY‐based delivery system that can progressively release QC to meet the demands of sustained release and prolonged efficacy. With the assistance of holotomographic microscopy, QC@GYs accumulated at the rim of the cell membranes, indicating the successful entry of NPs into the target cells. Our findings also revealed evidence of intercellular leakage upon coculture, which may contribute to increased permeability, allowing fluids and molecules to leak from the bloodstream into surrounding tissues. This observation underscores the potential implications of our research for understanding the mechanisms underlying pathological conditions characterized by compromised barrier function, such as in various diseases including AMD.

No cytotoxicity or systemic adverse effects were observed in vitro or in vivo. Previously, QC was shown to protect cardiomyocytes from oxidative stress‐induced mitochondrial dysfunction and rescue the jejunal mucosa from apoptotic cell death in streptozotocin‐induced diabetic rats.^[^ ^70^, ^77^ ^]^ In the present study, the conjugation of QC with GYs facilitated sustained drug release and longer retention time at the injured sites of the retina. We observed the antiapoptotic efficacy of QC@GYs in cultured RPE cells in vitro and in the RPE cell layer in a mouse model of dry‐type AMD. The QC@GY‐mediated antiapoptotic effects involved robust suppression of Bax expression and a decreased Bax/Bcl‐2 ratio. As evidenced by the suppression of microglial activation, QC@GYs also showed the ability attenuated neuroinflammation, putatively due to their QC@GY‐mediated antiapoptotic effects. OCT and ERG findings showed anatomical and functional improvements in the injured retina 7 d after NaIO_3_ administration. Because of their sustained release, the efficacy of QC@GYs lasted for >14 d.

## 4 Conclusion

In summary, we demonstrated a novel approach utilizing in situ ophthalmic tethered GY as both an ocular retention agent and reservoir for QC, aiming to address the challenge of low‐eye fundus drug retention. The unique cavity texture of the GYs enabled a loading efficiency of more than 40% for QC, with sustained release over a period of 15 d. QC@GY exhibited high biocompatibility, facilitating the disruption of cell–cell tight junctions (TJs) and efficient particle penetration. In an AMD model induced by NaIO_3_, QC@GY exhibited protective effects against NaIO_3_‐induced damage to RPE cells in vivo. This was achieved through a combination of ophthalmic tethering retention and sustained QC release. Furthermore, QC@GY treatment maintained a normal retinal structure, preserved ERG responses, and effectively mitigated the formation of drusen deposits and neuroinflammation in NaIO_3_‐injured retinas. Overall, our findings suggest that ophthalmic tethered QC@GY is a promising therapeutic strategy for the treatment of eye fundus diseases, offering prolonged drug release and enhanced efficacy in preserving visual‐retinal health.

## 5 Experimental Section

5.1

5.1.1

##### Synthesis and Modification of GYs

The preparation of GY involved a seeded growth method utilizing cubic silver chloride (AgCl) as a sacrificial template, on which gold wires were selectively deposited. Initially, cubic AgCl was synthesized by mixing 70 μL of AgNO_3_ solution (100 mM m) with 110 μL of HAuCl_4_ (40 mM m) solution. This mixture was then added to 4.5 mL a 100 mM m PVP solution by stirring. Subsequently, 160 μL of 28 mM m hydroquinone solution was slowly added to this solution at room temperature, avoiding light exposure. The solution underwent a rapid color change from light black to brown within 3 h. To eliminate the AgCl cores, the solution was washed three times with NH4OH. The resulting purified GY was dispersed in water for further use.

##### Synthesis of GNPs

GNPs were synthesized using the method described by Cheng et al. A mixture of 99 mL of water and 1 mL of HAuCl4 solution in a 250 mL beaker was stirred continuously, 50 mL of NaBH4 (0.1 mg) was slowly added, and 99 mL of water and 1 mL of HAuCl4 solution were introduced slowly. The solution was stirred overnight to complete the reaction.

##### Preparation of GNPs and QC Loading

A round‐bottom flask equipped with a reflux condenser was used to vigorously boil and stir 50 mL of aqueous trisodium citrate solution (4 mM m). Subsequently, 250 μL of 200 mM m HAuCl_4_ was quickly added, and the solution was boiled for an additional 15 min. During this period, the color of the solution shifted from pale yellow to purple and then to wine‐red. To load the QC, 1 mg of GNPs was dispersed in 1 mL of ethanol, and then 1 mg of QC was added to the ethanol dispersion of the GNPs. The solutions were sonicated for 15 min, followed by a 24 h period without agitation.

##### Conjugation of QC with GY

The nanoconjugated system was synthesized by conjugating QC to GY at a concentration of 5 mg mL^−1^ for 45 min under continuous stirring. Subsequently, to purify the QC@GY NPs, an ultracentrifugation step was carried out at 25024 g for 40 min at 15 °C. Further characterization and biological experiments were conducted using intense red loose pellets of QC@GYs obtained from the purification process.

##### Quantification of QC in GNP by UV‐Visible Spectroscopy

QC@GY, GNP, and QC absorption were measured using a UV–vis spectrophotometer (JASCO dual‐beam spectrophotometer [Model V‐570]) between 800 and 200 nm with a resolution of 1 nm. The spectroscopic analysis of UV–vis light was utilized to construct a standard curve of QC (5–25 μg mL^−1^ in GNP supernatant) by plotting the absorbance of QC (*λ*_max_ ≈ 379.5 nm) at their respective concentrations (μg mL^−1^). The standard curve and absorbance of the QC@GY supernatant were used to calculate the amount of unconjugated QC in the supernatant and the percentage of attached QC.

##### Transmission Electron Microscopy Studies

TEM was used to examine the size, shape, and morphology of NPs using Tecnai G2 F30 S‐Twin Microscope operated at 100 kV.

##### Cell Viability Assay

AlamarBlue assay (Bio‐Rad) was used for evaluating cell viability and proliferation. Cells were washed with phosphate buffered saline (PBS) and complete medium with 10% AlamarBlue (BioRad) was added to each well and incubated under standard incubation conditions for 5 h according to the manufacturer's protocol. Finally, the absorbance was measured by the reading at 540 nm on a microplate spectrophotometer (Tecan, Männedorf, Switzerland).

##### Live‐Cell Imaging

The refractive indexes of the QC@GY localization within ARPE‐19 cells were measured, and 3D index images were constructed using the Nanolive commercial microscope (Nanolive SA, Lausanne, Switzerland). Holograms were recorded using a digital camera by combining the beam that passed through the sample with the reference beam. The rotation was performed for 24 h, capturing 100 holograms per rotation. High‐resolution images of each sample layer were obtained using a synthetic aperture and multiple‐viewpoint holography. 96 z‐stacks images were displayed in grayscale for a depth of field of 30 μm. The raw data were transferred into FIJI, an open‐source platform for analyzing biological images.

##### Viability Analysis

The viability of cell cultures treated with QC@GY at various concentrations was assessed on a flow cytometer (BD Accuri C6 flow cytometer, Becton Dickinson, CA, USA) using FITC‐conjugated annexin V and PI. Analyses were conducted in accordance with the manufacturer's instructions. The RPE cells were trypsinized and centrifuged at room temperature after being trypsinized. After aspirating the supernatant from the cell pellet, the supernatant was resuspended in 200 mL of annexin V‐binding buffer containing annexin V‐FITC (1 L mL^−1^) and incubated at room temperature for 25 min. The suspension was then incubated for 5 min at room temperature with PI dye (10 g mL^−1^) before being analyzed using a flow cytometer. Phosphatidylserine relocalized to the outer surface of the plasma membrane during the early stages of apoptosis, where it can be detected with fluorescently labeled annexin V. Annexin V‐ and PI‐negative cells were defined as viable cells. Apoptotic cells were defined as annexin V‐positive and PI‐negative. Necrotic cells had permeable membranes, which enabled PI to pass through the membranes and stain double‐stranded DNA.

##### Western Blot Analysis

The ARPE‐19 cells were lysed in RIPA buffer containing 1X protease inhibitor cocktail (Merck Millipore, Burlington, MA, USA). Bradford assay was used to determine the protein concentrations. The cell lysate (50 g) was subjected to electrophoresis in 12% sodium dodecyl sulfate polyacrylamide gel and then transferred to PVDF membranes. The membranes were incubated overnight at 4 °C in TBST with 5% skimmed milk with the following primary antibodies: Bcl‐2 (Cell Signaling Technology, Danvers, MA, USA), Bax (Cell SignalingTechnology), caspase 3 (Sigma‐Aldrich, St. Louis, MO, USA), PARP (Cell Signaling Technology), c‐PARP (Cell Signaling Technology), and GAPDH (Cell Signaling Technology). Next, membranes were washed three times with TBST and incubated with HRP‐conjugated secondary antibodies including goat antimouse IgG (Invitrogen/Thermo Fisher Scientific, Waltham, MA, USA) and goat antirabbit IgG (Invitrogen/Thermo Fisher Scientific) for 1 h at room temperature. Using the ChemiDoc XRS Imaging System (Bio‐Rad, Hercules, CA, USA), protein bands were detected using the chemiluminescence system (ECL kit). Bands were analyzed and quantified by the ImageJ analysis software (NIH, Bethesda, MD, USA).

##### Experimental Model of AMD

We followed the guidelines of the Association for Research in Vision and Ophthalmology (ARVO) Statement on Use of Animals in Ophthalmic and Vision Research and obtained approval from the Institutional Animal Care and Use Committee of Taipei Veteran General Hospital (IACUC), approval number 2020‐038 (1 January 2020). Healthy male C57BL/6 J mice, 8 weeks old and weighing ≈20 g, were purchased from LASCO and housed under standard conditions of a 12:12 h dark–light cycle, with access to standard rodent chow and water ad libitum. Three mice were used for each experimental group, and all mice were treated in accordance with the guidelines set forth by ARVO. The induction of AMD was conducted following the procedures described previously.^[^ ^14^, ^52^, ^53^ ^]^ Fresh NaIO_3_ solution was prepared by dissolving solid NaIO_3_ (#71 702, Sigma‐Aldrich (St. Louis, MO, USA)) into 0.9% sterile sodium chloride just before injection into the mice. 30 mg kg^−1^ NaIO_3_ was intraperitoneally injected into the mice. Retinal structure and functions were assessed using electroretinogram (ERG) and OCT at the baseline (day 0) and different days postinjection

##### Histological Toxicity Assessment

The mice were euthanized using the cervical dislocation method and all major organs (heart, liver, spleen, lung, and kidneys) were harvested. The organs were weighed and fixed for 24 h in 4% paraformaldehyde (Sigma‐Aldrich). All tissue specimens were then dehydrated using a Leica TP1020 tissue processor (Leica Microsystems, Wetzlar, Germany) and embedded in paraffin according to the standard procedure. Longitudinal 8 μm‐thick sections were obtained, placed on microscope slides (Thermo Fisher Scientific) and stained with H&E. The histological images were obtained using an Olympus BX46 microscope (Olympus Corporation, Tokyo, Japan).

##### In Vivo Study of QC@GY

Mice were divided into five groups: a control group, NaIO_3_ group, QC group, GY group, and QC@GY group. QC@GY mice were administered NaIO_3_ by intraperitoneal injection for 1 day following the administration of QC@GY (100 mg kg^−1^) through intravitreal injection. The healthy control group received intraperitoneal placebo injections and the NaIO_3_ group received intravitreous injections of placebo. H&E staining and real‐time images were used to analyze the retinal structure. The retina was collected in order to collect proteins for further study.

##### Electroretinogram Testing, Fundoscopy, and Optical Coherence Tomography (OCT)

Five groups of mice: a control group, NaIO_3_ group, QC group, GY group, and QC@GY group were examined at two time points (day 1 and day 7). Tropicamide 1% and phenylephrine 2.5% eye drops were used to dilate pupils after overnight dark adaptation. After that, mice were injected with 0.01 mL g^−1^ body weight of anaesthetic solution containing 100 mg mL^−1^ ketamine and 20 mg mL^−1^ xylazine. Following this, binocular ERG testing was conducted using the Lab Cradle (Diagnosys LLC). DA (scotopic) intensity series ERGs were performed (9 steps; 0.0025–10 cd m^−2^) followed by a 10‐min light adaptation (LA; 30 cd m^−2^). For each step of the DA ERG, both a‐wave, and b‐wave amplitudes were measured. An a‐wave amplitude was measured from the baseline to the trough of the first negative waveform, whereas a b‐wave amplitude was measured from the trough of the a‐wave to the peaks of the subsequent positive waveform.

After ERG testing, eye fundus and retinal OCT were performed using Phoenix MICRON‐III. Multicontrast OCT image of the mouse retinal structure was obtained after anesthesia with a mixed solution of Zoletil (50 mg kg^−1^) and Ropum (10 mg kg^−1^). Eye‐fundus photographs were acquired with a retinal imaging camera (Nikon, Shinagawa, Tokyo, Japan). Images were captured by fundoscopy and OCT in the exact area of the retina surrounding the optic nerve.

##### Immunocytochemistry

Mouse eyes were fixed in 1% paraformaldehyde in 0.1 m phosphate buffer at room temperature for 1 h, and immunohistochemistry of frozen eye sections was performed as described previously.^[^ ^55^ ^]^ The primary antibodies against rhodopsin (#AB221664; Abcam, Cambridge, UK; dilution 1:100) and RPE65 (#AB13826; Abcam; dilution 1:1000) were used. The secondary antibodies were Alexa Fluor 594‐conjugated antirabbit or Alexa Flour 488‐conjugated antimouse (Invitrogen/Thermo Fisher Scientific; dilution 1:500). DAPI nuclear counterstain (Abcam) was used to identify the nuclei, and images were acquired using a confocal microscope. The sections were mounted in Vectashield mounting medium with DAPI (Vector Laboratories, Burlingame, CA), and images were acquired using a confocal microscope.

##### Histological Assessment of the Retina

The retina samples were fixed in 4% paraformaldehyde (Sigma, Saint Louis, MO, USA) overnight and then were rinsed in 1 × PBS. Fixation, dehydration, clearing, infiltration, and embedding were conducted according to Bio‐Check Laboratories’ protocols. The retina samples were sectioned into 3 μm slices using a vibratome (Leica, Buffalo Grove, IL, USA), followed by H&E staining. The retinal and choroidal structures were examined under a light microscope (Olympus America, Melville, NY, USA) to determine its morphology. The thickness of the retina and choroid, defined as the distance through ILM, retinal GCL, IPL, INL, OPL, ONL, IS/OS layer of photoreceptors, RPE layer, BM, and CC, was measured using Image J (NIH, Bethesda, Maryland, USA).

##### In Vivo Sustained Release Study

To investigate the capability of sustained drug release in vivo, we used Cy5.5 dye (Cytiva, 28 902 160) to mimic the release of QC. The dose administered was consistent with previous in vivo studies. Cy5.5‐labeled GY was injected into the mouse eyeball for a total of 12 days. Subsequently, IVIS images were captured to evaluate the release profile by detecting the Cy5.5 fluorescence signal.

##### Statistical Analysis

Descriptive statistics were performed using the Prism software package (PRISM 10, GraphPad Software, USA). The obtained data were the original data without normalization. Data were expressed as means ± standard deviation in the analysis of a minimum of triplicate independent experiments (*n* ≥ 3). Significance levels denoted as n.s. mean nonsignificant; *p < 0.05, **p < 0.01, ***p < 0.001, and ****p < 0.0005 were determined through one‐way and two‐way ANOVA with Tukey's multiple‐comparison test.

## Conflict of Interest

The authors declare no conflict of interest.

## Supporting information

Supplementary Material

## Data Availability

The data that support the findings of this study are available from the corresponding author upon reasonable request.
