# Supplementary material for: Ophthalmic Tethered Gold Yarnball‐Mediated Retained Drug Delivery for Eye Fundus Disease Treatment
Source: Small Sci. 2024 Jun 27;4(8):2400095. doi: 10.1002/smsc.202400095 (PMC11934989; doi:10.1002/smsc.202400095)
Supplement: Supplementary file 1 — Supplementary Material [file SMSC-4-2400095-s001.pdf]

Supporting Information

Ophthalmic Tethered Gold Yarnball-Mediated Retained Drug Delivery for Eye-fundus Disease Treatment

Shih-Jie Chou[1,2], *Yi-Ping Yang*[1,2], *Min-Ren Chiang*[3], *Chih-Ying Chen*[1,2], *Henkie Isahwan Ahmad Mulyadi Lai*[1,2], *Yi-Ying Lin*[1,2], *You-Ren Wu*[1,2], I-Chieh Wang[1,2], *Aliaksandr A. Yarmishyn*[1,2], Guang-Yuh Chiou[4], *Tai-Chi Lin*[5,6], *De-Kung Huang*[5,6], *Shih-Jen Chen*[5,6], *Yueh Chien*[1,2,*], *Shang-Hsiu Hu*[3,*], *Shih-Hwa Chiou*[1,2,5,6,7,*]

[1] Department of Medical Research, Taipei Veterans General Hospital, Taipei 112201, Taiwan
2 Institute of Pharmacology, College of Medicine, National Yang Ming Chiao Tung University, Taipei 112304, Taiwan
[3] Department of Biomedical Engineering and Environmental Sciences, National Tsing Hua University, Hsinchu, 300 (Taiwan)
[4] Department of Biological Science and Technology, National Yang Ming Chiao Tung University, Taipei 112304, Taiwan
[5] Department of Ophthalmology, Taipei Veterans General Hospital, Taipei 112201, Taiwan.
[6]School of Medicine, National Yang Ming Chiao Tung University, Taipei 112304, Taiwan
[7] Genomic Research Center, Academia Sinica, Taipei 115024, Taiwan.

E-mail: g39005005@gmail.com, shhu@mx.nthu.edu.tw, shchiou@vghtpe.gov.tw

Experimental section

In vitro enhanced penetration study of GYs

To evaluate the enhanced penetration of GY *in vitro*, the experiment was performed using a Transwell assay designed for particle penetration studies, using retinal pigment epithelial (RPE) cells in both the upper and lower layers of the Transwell insert. This experiment was designed to evaluate the barrier properties of the RPE cell layer to particulate matter. Initially, RPE cells were seeded on the upper side of the Transwell insert, while the same RPE cell population was seeded on the lower side. After incubation to allow cell attachment and monolayer formation, the Transwell insert was placed in a multiwell plate device. Particles of a specific size range and

concentration are then introduced into the upper chamber, simulating exposure to airborne particles. Quantitative analysis of particle penetration can be performed by techniques such as microscopy.

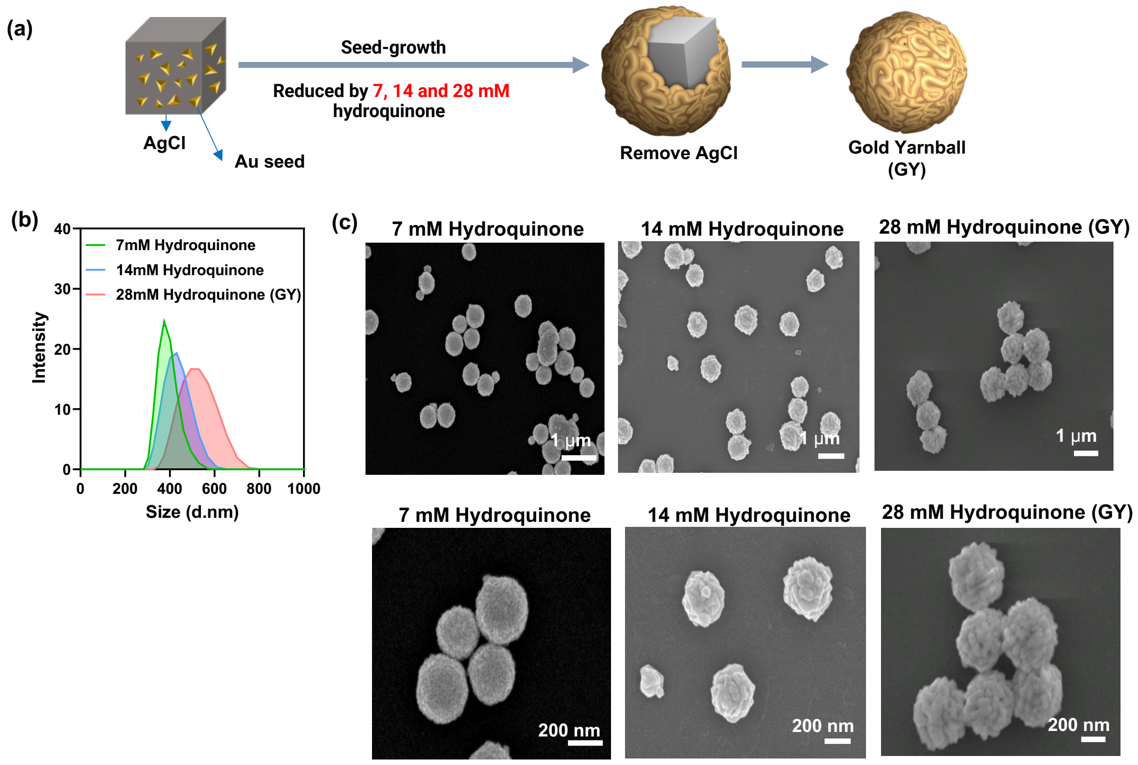

Figure S1. (a) Preparation a of quercetin-conjugated gold yarnball (QC@GY) nanoparticles with various concentrations of reduction agent (hydroquinone). (b) Size distribution. (c) Scanning electron microscopy (SEM) images of GYs with various concentrations of reduction agent.

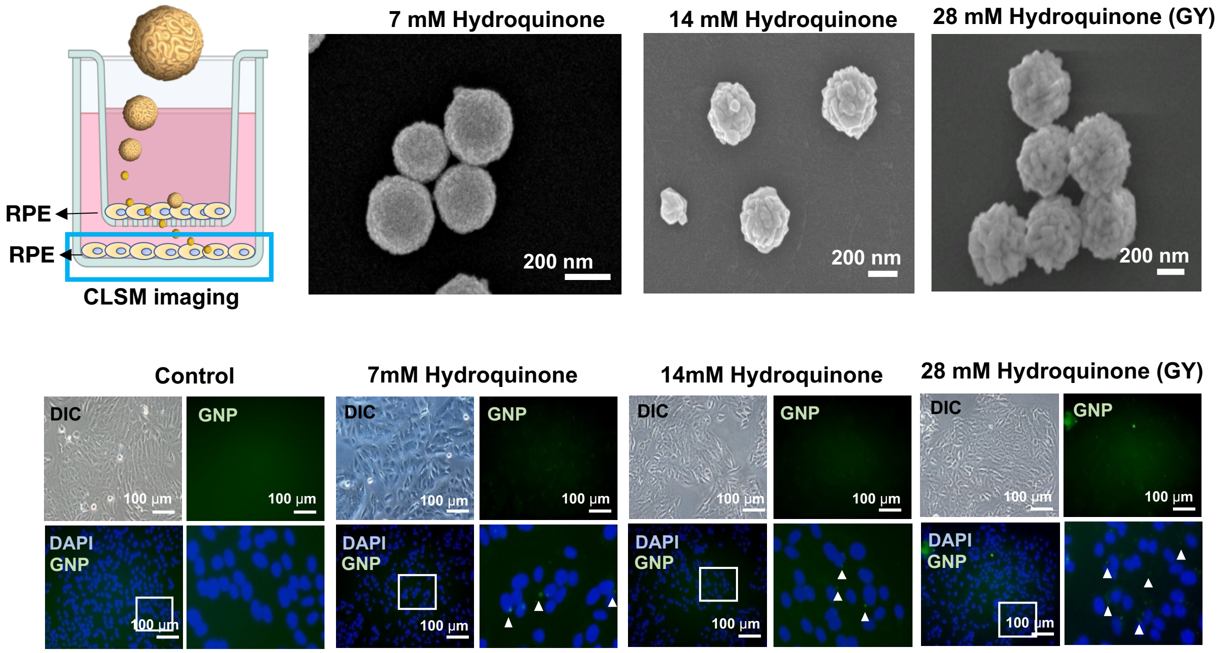

Figure S2. Transwell was used to assess the permeability of GY *in vitro* through retinal pigment epithelial (RPE) cells. The RPE cells were seeded in both the upper and lower layers of Transwell inserts, creating a dual-layered barrier model. The study aimed to investigate the enhanced permeability characteristics of GY particles. (Bottom) CLSM images of lower layers of Transwell inserts, indicating the penetration of particles.

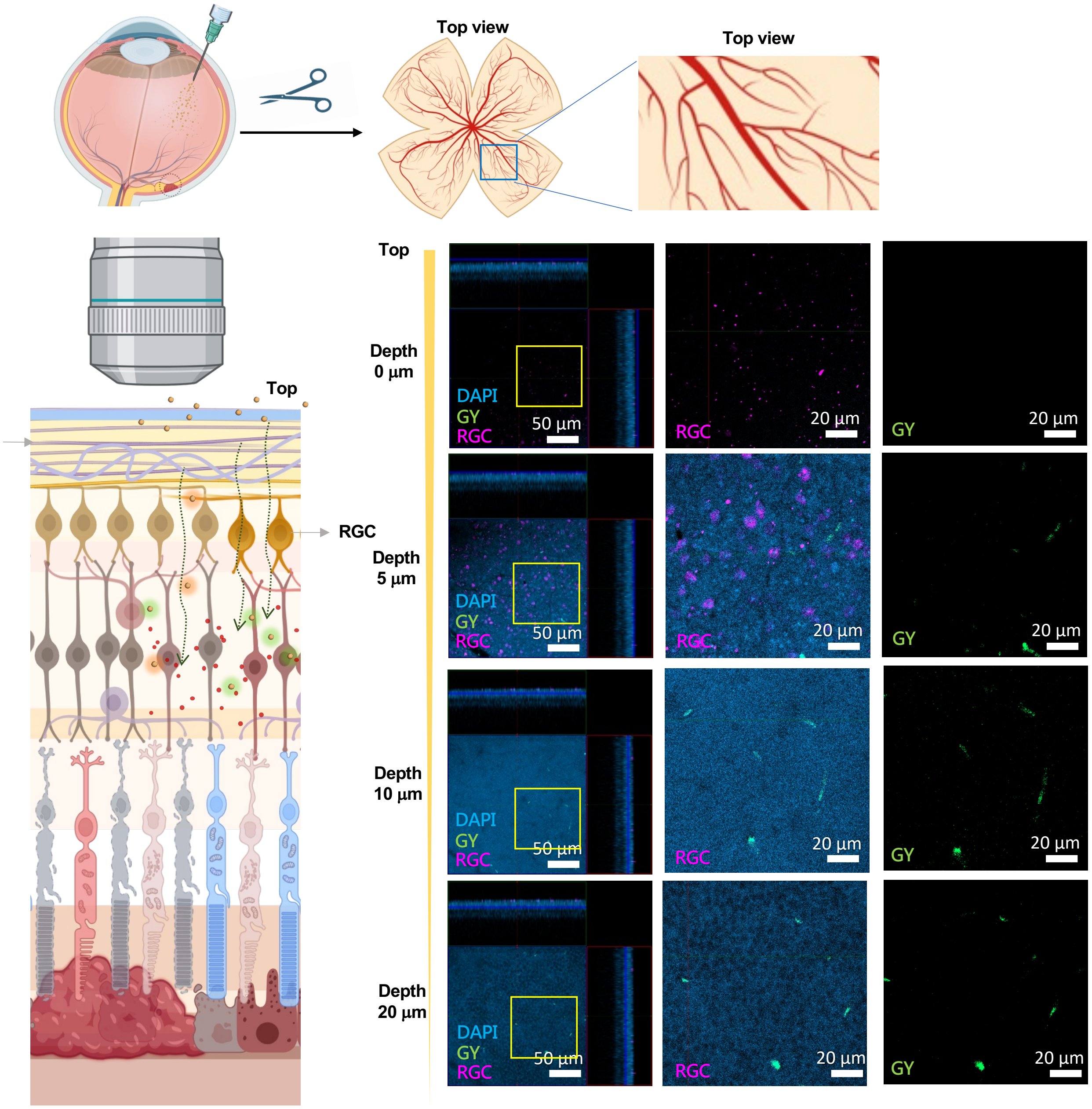

Figure S3. CLSM image depicting GY-treated retina. Signals from granules were observed beneath the retinal ganglion cell (RGC) layer, indicating effective penetration of the treatment.

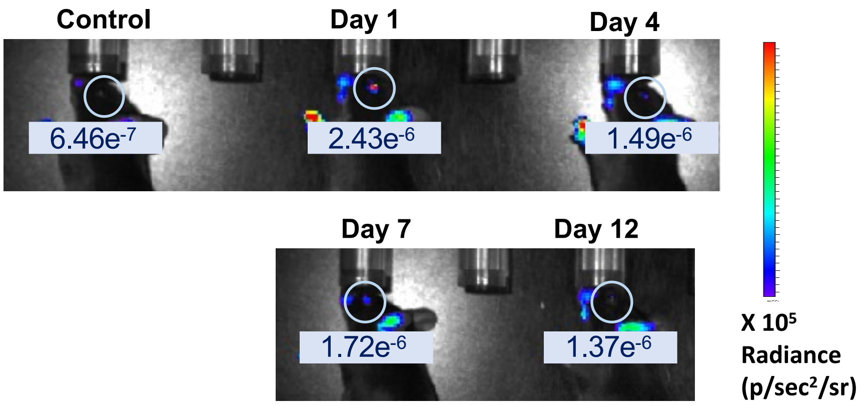

Figure S4. *In vivo* fluorescence images of major clearance organ of control, COF, dCOF and APON.

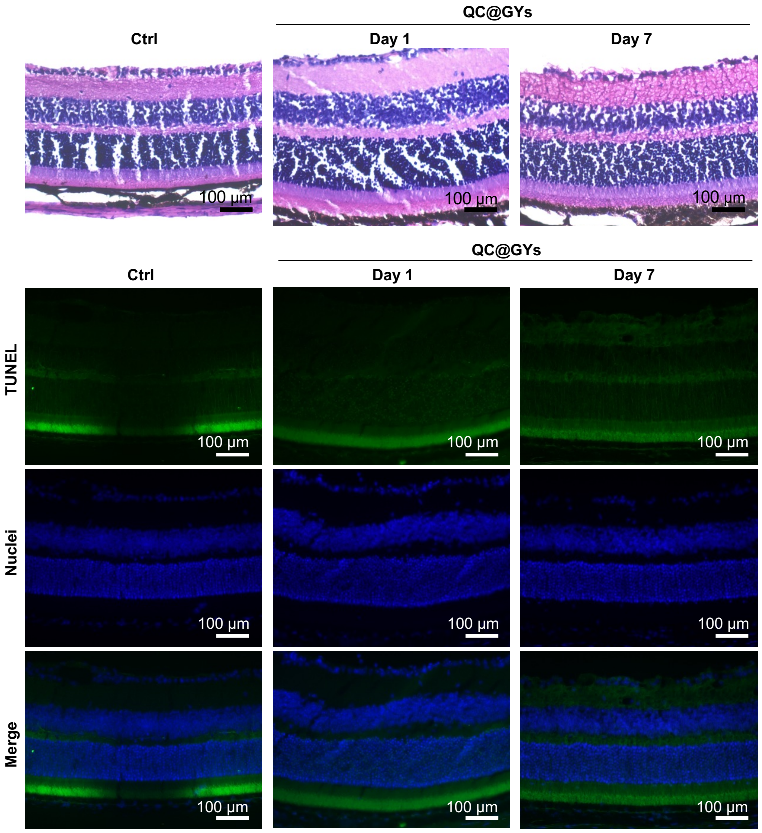

Figure S5. H&E staining and TUNEL imaging were performed following intravitreal injection of QC@GYs into the eyes of mice at 1 and 7 days post-injection.
